# Direct Processing of PVD Hard Coatings via Focused Ion Beam Milling for Microinjection Molding

**DOI:** 10.3390/mi14020294

**Published:** 2023-01-22

**Authors:** Holger Ruehl, Thomas Guenther, André Zimmermann

**Affiliations:** 1 Institute for Micro Integration (IFM), Faculty 7—Engineering Design, Production Engineering and Automotive Engineering, University of Stuttgart, Allmandring 9b, 70569 Stuttgart, Germany; 2 Hahn-Schickard, Allmandring 9b, 70569 Stuttgart, Germany

**Keywords:** direct processing, focused ion beam, injection molding, PVD, hard coating, DLC, CrN

## Abstract

Hard coatings can be applied onto microstructured molds to influence wear, form filling and demolding behaviors in microinjection molding. As an alternative to this conventional manufacturing procedure, “direct processing” of physical-vapor-deposited (PVD) hard coatings was investigated in this study, by fabricating submicron features directly into the coatings for a subsequent replication via molding. Different diamondlike carbon (DLC) and chromium nitride (CrN) PVD coatings were investigated regarding their suitability for focused ion beam (FIB) milling and microinjection molding using microscope imaging and areal roughness measurements. Each coating type was deposited onto high-gloss polished mold inserts. A specific test pattern containing different submicron features was then FIB-milled into the coatings using varied FIB parameters. The milling results were found to be influenced by the coating morphology and grain microstructure. Using injection–compression molding, the submicron structures were molded onto polycarbonate (PC) and cyclic olefin polymer (COP). The molding results revealed contrasting molding performances for the studied coatings and polymers. For CrN and PC, a sufficient replication fidelity based on AFM measurements was achieved. In contrast, only an insufficient molding result could be obtained for the DLC. No abrasive wear or coating delamination could be found after molding.

## 1. Introduction

Over the last decade, the current state of research in achievable lateral and vertical dimensions using polymer replication technologies, such as microinjection molding, has shifted more and more from the micron scale [1] towards the submicron, respectively, nanometer, scale [2]. This development has been driven by continuous advances of micro and nanofabrication technologies for surface texturing. Mold manufacturers have to carefully choose among technologies based on the working principles of mechanical machining, electrochemical etching, thermoelectric engraving and additive manufacturing in order to identify the most suitable one for manufacturing a structured mold tool, while considering the required surface geometry, shape and dimensions, the material to be processed and economic aspects [3]. As suitable structuring technologies for submicron structured tool making, beam-based methods such as direct laser writing with a femtosecond laser, lithography techniques, e-beam writing and focused ion beam (FIB) milling were identified by [3,4]. In [5], Holzer et al. utilized extreme-ultraviolet-interference lithography to create nanostructures in silicon, which were successfully replicated into cyclic olefin copolymer (COC). Calaon et al. used e-beam lithography followed by an etching of nanostructures into silicon, which were replicated onto a nickel shim [6]. The nanostructures were then molded into COC for a lab-on-a-chip. For optical applications, a Fresnel lens was FIB-milled into titanium mold inserts [7] and injection–compression-molded (ICM) [8].

Form filling and adequate demolding, already challenging in the molding of microfeatures, become even more delicate when replicating features at the submicron level. While the filling of cavities is attributed to the melt injection temperature and mold temperature [9,10,11], these replication-enhancing parameters also affect the demolding forces [12]. Acting forces in injection molding are the thermal contraction force related to the polymer, friction forces, adhesive forces and stiction, as well as a deformation force during the ejection stage [13]. All of these forces contribute to the demolding force, because of which the usage of simplified experiments and the development of predictive models to evaluate the demolding behavior is of great interest [14,15]. One possible solution to influence the form filling and demolding is the application of chemical-vapor-deposited (CVD) and physical-vapor-deposited (PVD) hard coatings onto the mold surface [16], which has been successfully applied by different studies. Bobzin et al. investigated the effects of conventional and variothermal processing for the replication of laser-machined 40 µm and 10 µm wide microgrooves, postcoated with CrAlN with varying Cr/Al ratios, whereas for the combination of uncoated molds and conventional molding, the 40 µm microgrooves were only partly filled, and the replication fidelity could be increased with coated molds and variothermal molding. For the 10 µm channels, however, clamping effects dominated the demolding, leading to distorted structures due to stretching. Focusing on the demolding behavior, Griffiths et. al. compared the demolding force of uncoated versus diamondlike carbon (DLC)-coated mold inserts for producing microfluidic chips [17]. The demolding force could be reduced by approx. 40% for ABS and 15% for PC compared to an uncoated mold insert.

In these presented studies, the coatings were deposited subsequent to the toolmaking, which is defined by Dumitru et al. as “indirect processing” [18]. In current research, lateral microstructure dimensions down to 10 µm are coated [19]. “Direct processing”, in which coatings are deposited first and then directly structured in a second step, could be an adequate solution to create coated mold tools containing submicron structured surfaces. In [20], the DLC coating deposited on the mold insert in [17], was direct-processed by plasma etching. By using this nanostructured DLC-coated tool, the demolding force of polypropylene could be reduced by 15.8%. However, for discrete structures, one might still be limited to the above-mentioned beam-based manufacturing technologies. These are, however, mostly used in materials science, with comprehensive studies on nanomachining discrete structures seeming to be the exception. The first investigations on the influence of different beam parameters to study the properties of tetrahedral hydrogen-free amorphous (ta-C) DLC coatings were carried out by Stanishevsky [21]. Via gas-assisted focused ion beam milling, redeposition effects could be reduced and the relatively low sputter yield of around 0.1 µm^3^/nC could be further increased [22]. Investigations about the FIB structuring of inorganic nonmetallic materials, such as TiN or CrN, are to the authors’ knowledge currently limited to cross-section milling for coating inspection [23,24] or investigations of material properties [25].

In this study, the direct processing of PVD hard coatings via focused ion beam milling is investigated in order to create submicron structures, which are then to be replicated using injection–compression molding. To begin with, the morphology and topography of different chromium nitride and diamondlike carbon thin films was characterized in terms of suitability for focused ion beam milling in mold making. Further FIB milling experiments were then carried out on each coating type. A test pattern consisting of different submicron features was FIB-milled choosing various beam parameters. The direct processed PVD hard coatings are then replicated onto polycarbonate (PC) and cyclic olefin polymer (COP). The replication fidelity of the molded submicron structures was evaluated using atomic force microscopy (AFM). The direct processed coatings were SEM-imaged to study wear effects after the molding experiments.

## 2. Materials and Methods

The scanning electron microscope (SEM) unit of a Helios NanoLab 600 FIB-SEM dual beam system (FEI Company, Hillsboro, OR, USA) was used for the characterization of the PVD hard coatings before and after FIB milling (Section 2.1) and subsequent to the molding experiments (Section 2.3). The FIB unit with a Ga-ion beam was used for the FIB milling experiments (Section 2.2).

For topography examinations, areal roughness measurements were conducted with a 3D optical profiler Zygo Nexview NX2 (Zygo Corporation, Middlefield, OH, USA). Areal roughness can be characterized in relation to a specific part’s function [26], which was maximum substrate smoothness in this study.

An AFM Veeco Dimension 3100 (Veeco Instruments Inc., Plainview, NY, USA) was used to measure the dimensions of the FIB-milled test pattern as well as of the replicated features in tapping mode.

### 2.1. Investigation of PVD Hard Coatings

The surface morphology of coatings, including defects, as well as the inherent surface topography, strongly depends on the coating material and used deposition technology. Wavy and rough surfaces are generally not suitable for creating submicron structures using FIB milling, that is, when the hills and dales of a surface are of higher lateral dimensions than the surface texture features itself or when the surface asperities around are higher or deeper than these features. For these reasons, different PVD hard coatings, provided by industrial manufacturers (M1–M4), were investigated first. A batch of six steel samples, all polished down to an equal roughness of Sa = 1.65 ± 0.25 nm, Sq = 2.2 ± 0.4 nm and Sz = 25.6 ± 12.2 nm, was coated either with different DLC ta-C or CrN coatings. Table 1 lists the samples, coating types, manufacturer nos. and applied PVD technology. Five coatings were deposited via arc deposition, which is widely offered in the industry. One CrN coating was deposited via high-power impulse magnetron sputtering (HiPIMS).

The coating morphology was then characterized with respect to texture and defects by using the SEM. The imaging was done for a 128 µm field of view (FOV). The areal roughness was measured for the same FOV before and after coating deposition. The generated data were evaluated by choosing operators in accordance with ISO 25178-3 [27]. Based on these results, a simple categorization into suitable or nonsuitable hard coatings regarding both direct processing via FIB milling and ICM was made in this study.

### 2.2. Focused Ion Beam Milling Experiments

As the focused-ion-beam-milled structures were to be replicated via molding, steel mold inserts for an injection–compression-molding tool were used as test substrates. After CNC-machining, a polishing machine Buehler Alpha 2 Speed (Buehler, Lake Bluff, IL, USA) was used for fine grinding and the subsequent polishing of the melt-facing surface prior to the PVD coating. The two mold inserts were then coated with one DLC ta-C and one CrN coating selected from Table 1.

**Figure 1:**
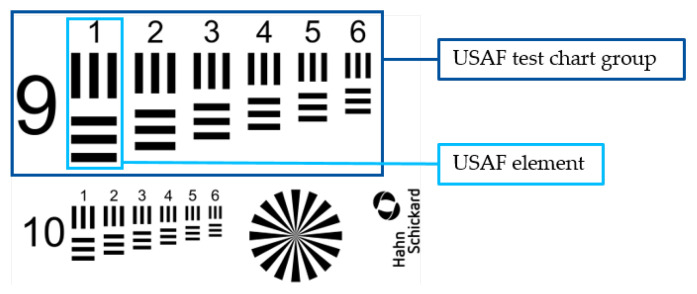
Test pattern design containing the USAF test chart groups 9 and 10, a Siemens star and logo.

A test pattern, shown in Figure 1, consisting of the USAF test chart groups 9 and 10 as well as a Siemens star was FIB-milled into the coatings. Each USAF group consists of six elements containing three horizontal and vertical rectangular bars. The width of one bar for each element and group is given in Table 2. The base width of each Siemens star spoke was equivalent to the bar width of USAF group 9 element 1.

For FIB milling, the ion acceleration voltage was set to 30 kV. The beam overlap was set to 50%. The dwell time t was set to 1 µs. Further milling parameters are provided in Table 3. The aperture beam current I (nA) and the number of passes n (-) were varied in order to investigate the influence of the ion beam diameter on the milled structure geometry while keeping the total ion dose constant. A total ion dose of 1337 pC/µm^2^ was impinged upon the hard coatings. The beam was rastered in a serpentine scan over the substrate.

After FIB milling, the SEM measurement tool was used to measure the lateral width of single test pattern elements. In addition, the test patterns were recorded with an AFM. For reference, the dimensions of the vertical middle bar of USAF group 10 element 1, FIB milled for I = 260 pA into the CrN, were measured. The AFM tip was rastered over the surface with a tip velocity of 197 nm/s for 16 lines at 512 measuring points/line.

### 2.3. Injection–Compression Molding Experiment

**Figure 2:**
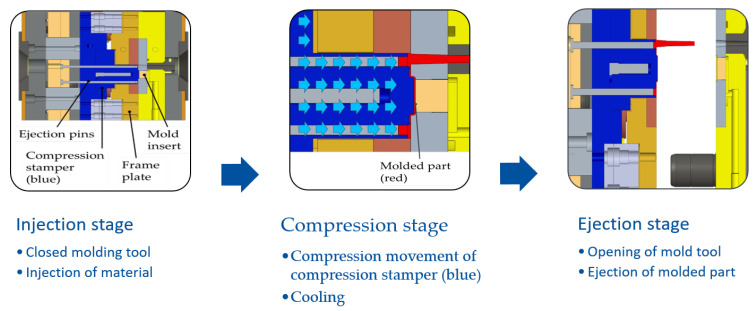
Mold tool and basic injection–compression molding’s processing sequence.

The replication of the submicron structures via injection–compression molding was conducted on an injection-molding machine ALLROUNDER 270 A (ARBURG GmbH & Co. KG, Loßburg, Germany). A microinjection module (ARBURG) was used for a more homogeneous melt plasticizing and a more precise injection volume dosage than the standard module. The fundamental components of the injection–compression-molding tool used, described in detail in [28], and the ICM processing sequence are shown in Figure 2. The molding tool cavity was closed via a frame plate prior to the injection of the plasticized melt. After filling of the cavity, the compression step was executed by moving the compression stamper (shown in blue in Figure 2) towards the structured mold insert, followed by cooling. In the last step, the mold tool was opened and the molded part was ejected. The molded part was a flat plate with 0.5 mm thickness and a rectangular area of 13.3 × 10.3 mm to integrate different micropatterns intended for optical applications. The plate was surrounded by a stepped assembly frame.

Polycarbonate LEXAN 133R (SABIC, Riyadh, Saudi Arabia) and COP ZEONEX 330R (Nippon Zeon K.K., Chiyoda, Japan) were chosen as molding materials. The polymer properties are given in Table 4. The ICM processing parameters are listed in Table 5.

The surface roughness of the polymer parts was measured on unstructured areas applying the same areal measurement conditions as used for the PVD hard coatings.

For the structured areas, an AFM scan of an entire test pattern was exemplarily made for a PC sample. To evaluate the replication performance, the height of the molded bar for USAF group 10 element 1 was evaluated and compared to the FIB-milled test pattern depth.

After the ICM experiments, the FIB-milled test patterns were again SEM imaged in order to investigate abrasive and adhesive wear as well as delamination effects on the hard coatings.

## 3. Results

### 3.1. Preselection of Hard Coatings for FIB Milling and Molding Experiments

**Figure 3:**
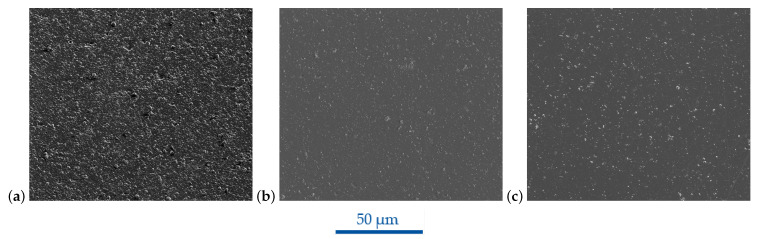
SEM images of tetrahedral hydrogen-free amorphous diamondlike carbon coatings provided by different manufacturers; (**a**) DLC ta-C 1 M1, (**b**) DLC ta-C 2 M1 and (**c**) DLC ta-C 3 M2 coating at FOV 128 µm.

Figure 3 shows the SEM images of the three investigated DLC ta-C coatings. Despite all of these coatings being of the same type and being deposited via arc vaporization, they differed in surface morphology. The DLC ta-C 1 showed a microscopically wavy surface morphology consisting of nanoscaled grains, unevenly covered with big droplets of agglomerated grains as well as craters. DLC ta-C 1 was neither usable for ion beam milling nor for microinjection molding of structured surfaces. Preliminary FIB-milling trials resulted in uneven lateral edges. In the case of molding structured surfaces with specific structures in order to provide an actual function, the roughness given in Table 6 was too high and was replicated onto the polymers, likely resulting in nonfunctional parts. Contrary to this finding, the DLC ta-C 2 and DLC ta-C 3 coatings replicated the initial surface morphology very well and were thus microscopically smooth. Initial microscratches from polishing were however still replicated, and the coatings were not completely defect-free. Protruding growth defects were randomly distributed over the entire surface. The areal surface roughness measurements in Table 6 underline these results. Both DLC ta-C 2 and DLC ta-3 provided low areal Sa and Sq values. By a mere optical evaluation, the DLC ta-C 3 seemed to have slightly less droplets, which might be the reason for the slightly lower Sz value. For these reasons, the DLC ta-C 3 coating provided the best choice among the three DLC films and was therefore chosen for the FIB milling and subsequent molding experiments.

**Figure 4:**
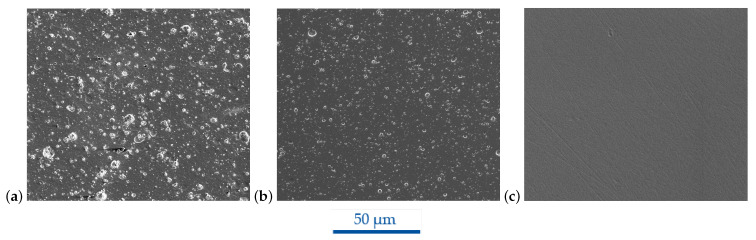
SEM images of CrN coatings provided by different manufacturers; (**a**) CrN 1 M3, (**b**) CrN 2 M4 and (**c**) CrN 3 M1 coating at FOV 128 µm.

The different CrN coatings are shown in Figure 4. The CrN 1 morphology exhibited the highest roughness of the inspected CrN coatings and was additionally equally covered with a high number of droplets. This finding is in accordance with the highest areal surface roughness result given in Table 6. For these reasons, CrN 1 was not taken into further consideration. The CrN 2 hard coating was polished after the arc deposition as part of the manufacturer’s standard procedure to remove droplets. It showed a relatively smooth morphology but was also covered with droplets over the entire area. Compared to CrN 1, the droplets were smaller, but in addition, circular craters could be found. Its surface roughness was lower compared to CrN 1, except the Sv value. CrN 3 was a smooth coating with dimpled texture. The initial polished substrate morphology was very well replicated, as it can be seen from the polishing scratches. However, single flake defects were still present in low amounts, which seemingly could not be completely avoided during deposition. Under the three investigated CrN coatings, CrN 3 showed the smallest roughness for all evaluated parameters. Due to the smoothest morphology and lowest roughness of all CrN coatings, the HiPIMS-sputtered CrN 3 was chosen and used for the FIB milling and subsequent molding experiments.

For simplification, these selected samples are referred to as DLC ta-C and CrN in the following.

### 3.2. Direct Processing via FIB Milling

**Figure 5:**
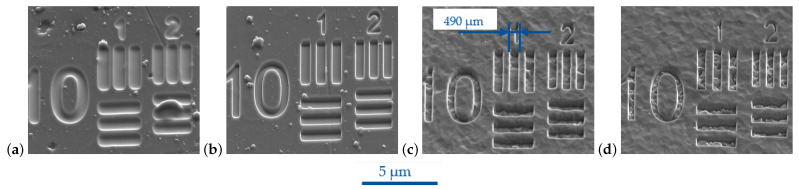
SEM images of USAF group 10 elements 1 and 2 FIB-milled into diamondlike carbon ta-C at beam currents (**a**) I = 260 pA and (**b**) I = 9 pA and into HiPIMS CrN at beam currents (**c**) I = 260 pA and (**d**) I = 9 pA.

The main findings of the FIB direct processing are exemplified by comparing the USAF group 10 elements shown in the Figure 5a–d. Preliminary results were already presented in [29].

Figure 5a shows the test pattern FIB-milled for I = 260 pA compared to I = 9 pA in Figure 5b. The feature integrity depended on the ion beam milling parameters as well as the substrate material itself. For both coating materials, smaller ion beam currents resulted in a higher resolution of lateral features, as seen when Figure 5a,b or Figure 5c,d are compared. This effect was more pronounced for the DLC, as can be derived from Figure 5a. For I = 260 pA, the corner radius of elements with a width <400 nm was relatively much larger compared to the width of the elements.

For the DLC ta-C, a rounding of outer and inner edges occurred, and the widths of the single element bars were slightly larger, as specified in Table 2. As a result, elements with a specified widths <400 nm were nearly blurred into each other. A slight redeposition of sputtered material could be detected with an increasing aspect ratio. Inside the FIB-milled ta-C structures, supersmooth surfaces were generated. Droplets locally hindered a homogeneous material removal, as it can exemplarily be seen for the horizontal bars of element 2 in Figure 5a.

**Figure 6:**
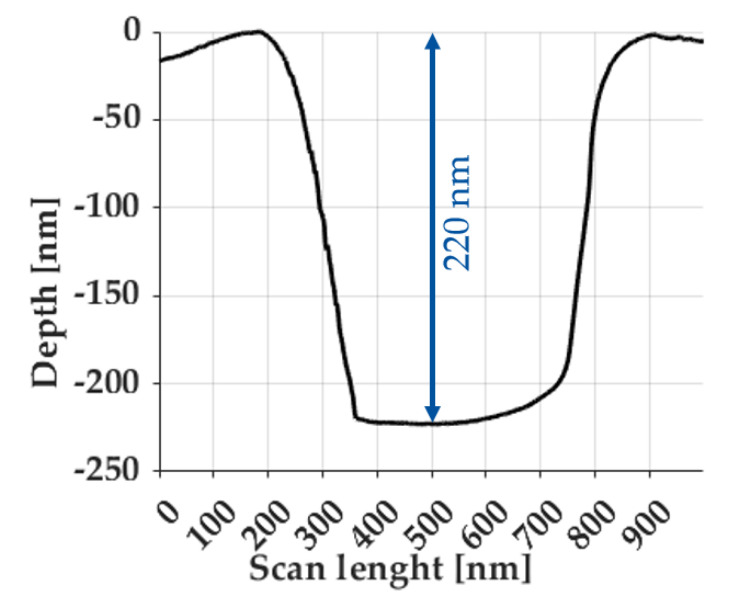
Profile depths of test pattern FIB-milled into CrN.

In opposite to the DLC ta-C, the rounding of edges was less pronounced for the CrN. The element widths measured with the SEM inspection tool were in the specified range of Table 3. The smallest measurable feature width was 63 nm, measured for the Siemens star spoke. In contrast to the DLC ta-C, a roughening inside the structures compared to the unprocessed areas was found. The roughening increased for smaller beam currents and ion beam diameters. In Figure 6, the AFM scan for the vertical middle bar of USAF group 10 elements FIB-milled into the CrN is given. A depth of 220 nm was achieved for the parameters from Table 3, resulting in aspect ratios in the range of 0.22:1 up to 0.8:1.

### 3.3. Injection–Compression Molding Results

**Figure 7:**
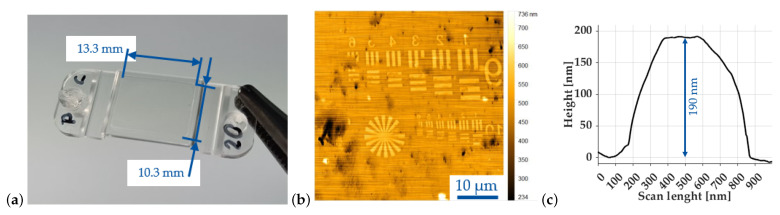
(**a**) Molded PC LEXAN 133R part with dimensions of the surface area containing replicated features; (**b**) AFM scan of full test pattern replicated from FIB-milled DLC ta-C hard coating; (**c**) profile height of replicated USAF 10 group 1 vertical bar onto PC for CrN.

One injection–compression-molded PC part with removed sprue is shown in Figure 7a. The areal surface roughness of the molded parts, measured on unstructured areas, is provided in Table 7. By comparing the roughness values with the mold roughness given in Table 6, it can be seen that there was only a minor difference between CrN-coated mold insert and the molded parts using this insert. For the DLC ta-C coated mold insert, the roughness of the PC parts were slightly higher in comparison. For the COP and the DLC ta-C, a completely different finding was made. The measured Sa and Sq values of the molded parts were three times higher compared to those of the DLC ta-C. Only the Sz values were within the measurement range of the corresponding coating.

Figure 7b shows an AFM scan of the replicated test pattern, molded from the direct-processed DLC ta-C with an ion beam current of 9 pA. It can be derived that the structures were replicated onto the polycarbonate, but that single features were only partly replicated as was the case for horizontal bars of the USAF group 10 and one spoke of the Siemens star. For the COP ZEONEX 330R, a replication of the test pattern from the DLC ta-C coating was not successful.

With the direct-processed CrN mold insert, a full replication both with PC and COP was achieved. The exemplarily executed AFM scan for the USAF 10 element 1 vertical middle bar molded onto PC is given in Figure 7c. The determined height was 190 nm. Thus, for this feature, an aspect ratio of 0.4:1 was achieved.

A SEM inspection of the direct-processed DLC ta-C coated mold insert subsequent to the COP molding revealed a high quantity of polymer residues distributed all over the entire hard coating. The residues also clogged the FIB-milled structures. In accordance with this finding, material breakouts were found on the equivalent molded parts during the surface inspection using the 50×-magnification objective of the 3D optical profiler. In contrast, no polymer residues were found on the CrN coating subsequent to molding. For both PVD hard coatings, no abrasive wear and delamination could be detected with the SEM.

## 4. Discussion

The surface quality of a coating is determined by several factors such as the initial substrate roughness, the substrate arrangement in the coater, the PVD deposition technology and the coating parameters including prior ion etching for substrate cleaning [30]. For this reason, surface coatings provided by industrial manufacturers can differ significantly in morphology and topography and therefore show varying suitability for both direct processing via FIB milling and polymer replication. DLC ta-C and CrN hard coatings deposited via arc deposition and sputtering were evaluated in this study. Due to the optically supersmooth roughness of the steel substrates, the influence of the substrate topography was assumed to be negligible in this study. All of the investigated coatings in this study were supplied by industrial manufacturers. A clear statement regarding the causes for the appearance of the different coatings can therefore be hardly given, but some aspects possibly having influenced the quality of the coatings are discussed in the following. Residuals from substrate pretreatment still might cause a growth of defects [31]. For arc deposition, the formation of macrodroplets accumulating during the deposition is correlated with the melting temperature of deposition targets and substrate configuration [32]. By supplying nitrogen into the coating chamber, a nitride layer forms on the target, which affects the melting temperature of the target. In case of CrN deposition, the melting temperature of Cr is lower compared to a nitrided Cr cathode target. The formation of droplets could be the reason for the much higher Sz and Sp roughness values of the arc-deposited coatings compared to the HiPIMS CrN. Since this might be a limiting factor for many applications of PVD hard coatings, different solutions for reduced droplet formation have been developed [33]. Compared to the manufacturers M1–M3, M4 used mechanical polishing subsequent to the coating process to abate the number of droplets. By this method, some single droplets were torn out, due to the poor bond between the coating matrix and the droplets [34]. However, craters were formed as a result of the polishing. This could also be the reason for the higher Sv value of CrN 2 compared to CrN 1. The HiPIMS CrN coating showed a supersmooth surface, containing almost no notable defects compared to the relatively large surface area, which is in good accordance with results of previous research [35,36].

The brief investigation of the PVD hard coatings revealed that mold-making manufacturers have to carefully choose a vapor deposition technology and coating material for its subsequent direct processing. Hard coatings deposited by sputtering technologies seem promising, because smooth, defect-free surfaces are needed for precise direct processing using micro- and nanomanufacturing technologies, as it was the case in this study. Defects, such as droplets occurring in arc deposition, result in locally nonuniform material removals. Prior to the coating deposition, the mold tool to be structured must be polished to optical roughness quality to limit the influence of the initial roughness to the coating morphology and roughness.

Based on the findings in the FIB-milling experiments, it can be stated that structure integrity and the roughness of the milled test patterns depend both on the substrate material properties and FIB milling parameters. For both inspected PVD hard coatings, lower ion beam currents, resulting in smaller beam diameters, could mill submicron structures more precisely. On the other hand, decreasing the beam diameter increased the processing time. The redeposition of already sputtered atoms was reduced due a lowered sputtering yield per scan with every additional scan repetition [37]. The rounding of edges might be caused by the Gaussian beam profile of ion beams. DLC ta-C coatings are of amorphous microstructure. It was found that the FIB-milled structures were slightly wider as specified, which is in accordance with the milling results of Stanishevsky [21] and which could be caused by a homogeneous isotropic sputtering. For very small distances below 400 µm of single pattern elements, additional proximity effects could have negatively amplified this sputtering behavior [38] for the DLC ta-C as well as for CrN. In contrast to amorphous carbon coatings, CrN coatings deposited by HiPIMS, are of crystalline microstructure [39]. Different FIB sputtering yields for different crystal directions and associated channeling might have caused the roughening of milled CrN [40].

In summary, both investigated PVD hard coatings could be direct-processed via FIB milling, creating submicron test patterns into hard coatings. It was found that both coating morphologies, including defects, surface roughness, the grain microstructure, as well as the FIB processing parameters, directly affected the sputtering yield, the dimensional accuracy and the achievable aspect ratios of the milled structures. Further experiments will concentrate on the sputtering yields of different PVD hard coatings, by considering their grain microstructures along with an extended variation of FIB parameters.

The micro ICM results were evaluated by surface roughness measurements of the polymer parts on unstructured areas and by AFM measurements of the replicated test patterns. The roughness of the PC and COP parts were equal to the CrN coating surface roughness. For the coating–polymer combination of DLC ta-C and PC, the areal roughness results were slightly higher but within acceptable tolerances. For all of these three cases it could be stated that the coating surfaces were well replicated onto the polymers, while also including inherent defects. The AFM scans showed that the test patterns could be fully replicated for the direct-processed CrN with both polymers. A replication height of 190 nm was achieved compared to a mold depth of 220 nm, providing a good replication fidelity of approx. 86%. This result seemed sufficient for the investigated molding parameters, since these parameters were not varied. In future experiments, an improved filling might be achieved by choosing polymer types with higher melt flow rates, which are then to be studied with an extended variation of the molding parameters. A further improvement of the replication fidelity might be obtainable with variothermal molding [19]. Demolding issues were only found for direct processed DLC ta-C. While with PC, the test patterns could be partly replicated, a high surface roughness and no replication of the test pattern was found for the COP parts. A closer inspection of both polymer parts and mold inserts revealed polymer residues on the DLC ta-C. These residues were torn out of the polymer parts, leaving defects, which increased the polymers’ surface roughness. A high adhesion between COP and DLC ta-C, leading to sticking and a material break out of the solidified polymer parts, might be the reason. Hence, different adhesion tendencies might be found for different PVD hard-coating–polymer combinations [14].

A challenging aspect for molding submicron features remains the final measurement. In this study, the height and depths of the created submicron structures could be evaluated by AFM measurements in order to determine the replication fidelity. Due to the AFM tip shape, a limitation is, however, the measurement of lateral submicron dimensions, especially for features with steep side walls as it was also reported in [41].

The final inspection of the FIB-milled test patterns in DLC ta-C and CrN showed no abrasive wear. Per polymer and coating type, around 100 parts were molded. For a full statement on the abrasive wear of the milled structures as well as on the adhesive wear between the mold insert and PVD coating causing delamination, long-term tests with several 1000 molding cycles must be made as done in [42], supported by simulations.

## 5. Conclusions

In this study, it was shown that the direct processing of PVD hard coatings via FIB milling was suitable to create submicron-sized structures which could be molded onto PC and COP. The main findings are:

- Mold making manufacturers have to carefully choose the deposition technology and coating material. For a direct processing of such coatings, in order to create submicron structures, the HiPIMS sputtering technology seems to be promising;
- In the direct processing of hard coatings using FIB milling, the sputtering yield of the material and the dimensional accuracy of milled features is dependent on the FIB milling parameters as well as on the grain microstructure and the surface roughness of the respective coatings;
- With ICM, direct-processed submicron structures with a dimensional height of 190 nm corresponding to an aspect ratio of 0.4:1 could be successfully replicated. The replication fidelity and demolding behavior of submicron-sized surface features depends on the coating–polymer material combination.

## Figures and Tables

**Table 1:** PVD hard coatings investigated in this study.

Sample No.	Coating Type	Manufacturer M and No.	PVD Technology
1	DLC ta-C 1	M1	Arc
2	DLC ta-C 2	M1	Arc
3	DLC ta-C 3	M2	Arc
4	CrN 1	M3	Arc
5	CrN 2 (post-polished)	M4	Arc
6	CrN 3	M1	HiPIMS

**Table 2:** Bar width per group element in (nm).

Group	Element 1	Element 2	Element 3	Element 4	Element 5	Element 6
9	980	873	778	693	617	550
10	490	437	389	346	309	275

**Table 3:** FIB milling parameters used in this study.

**I (pA)**	260	90	46	26	9
**n (-)**	1130	1948	2569	2761	6068

**Table 4:** Material properties of polycarbonate LEXAN 133 and COP ZEONEX 330R.

Properties	PC LEXAN 133R	COP ZEONEX 330R
Density (g/cm^3^)	1.20	0.95
Melt flow rate (g/10 min)	n.a.	11 (260 °C/ 21.18 N)
Melt volume rate (cm^3^/10 min)	3 (300 °C/1.2 kg)	n.a.
Transmission (%) (thickness (mm))	88–90 (2.54)	92 (3)
Refractive index	1.586	1.509

**Table 5:** Injection compression molding parameters for PC Lexan 133R and COP Zeonex 330R.

Parameter (Unit)	PC LEXAN 133R	COP Zeonex 330R
Injection temperature (°C)	350	260
Mold temperature (°C)	80	60
Injection pressure (MPa)	2000	
Holding pressure (MPa)	100	
Injection time (s)	0.21	0.26
Closing delay (s)	0.22	0.25
Compression force (kN)	45	
Compression time (s)	1	

**Table 6:** Areal surface roughness results of investigated PVD hard coatings at FOV 128 µm. The coatings highlighted in grey were selected for subsequent direct processing experiments.

Coating	Sa (nm)	Sq (nm)	Sz (nm)	Sp (nm)	Sv (nm)
DLC ta-C 1	30.55 ± 3.55	59.3 ± 8.00	1591.95 ± 109.15	773.20 ± 66.60	−818.80 ± 42.60
DLC ta-C 2	3.40 ± 0.20	6.95 ± 0.35	363.90 ± 98.8	173.20 ± 101.1	−216.40 ± 28.00
DLC ta-C 3	4.10 ± 0.20	6.34 ± 0.54	349.20 ± 118.60	104.60 ± 44.50	−248.65 ± 78.15
CrN 1	30.55 ± 2.35	81.25 ± 10.65	2688.65 ± 641.45	1855.40 ± 668.3	−666.85 ± 193.15
CrN 2	10.80 ± 4.10	30.85 ± 5.65	1180.80 ± 162.40	561.10 ± 116.50	−701.55 ± 127.65
CrN 3	5.05 ± 0.35	6.76 ± 0.76	179.15 ± 104.45	128.75 ± 106.75	−97.90 ± 49.80

**Table 7:** Areal surface roughness results of polymer parts measured at FOV 128 µm on unstructured areas.

Polymer Type and Hard Coating	Sa (nm)	Sq (nm)	Sz (nm)
PC and DLC ta-C	8.40 ± 1.40	13.75 ± 1.45	404.75 ± 18.05
COP and DLC ta-C	13.35 ± 0.45	19.50 ± 4.1	430.00 ± 50
PC and CrN	3.90 ± 0.30	5.1 ± 0.40	85.4 ± 0.40
COP and CrN	4.25 ± 0.55	5.75 ± 0.95	117.8 ± 62.60

## Data Availability

Not applicable.

## Abbreviations

The following abbreviations are used in this manuscript:

AFM	Atomic force microscopy
COC	Cyclic olefin copolymer
COP	Cyclic olefin polymer
CVD	Chemical vapor deposition
DLC	Diamondlike carbon
FIB	Focused ion beam
FOV	Field of view
HiPIMS	High-power impulse magnetron sputtering
ICM	Injection–compression molding
PC	Polycarbonate
PVD	Physical vapor deposition
SEM	Scanning electron microscope
ta-C	Tetrahedral hydrogen-free amorphous carbon film
